# Transcranial direct-current stimulation combined with attention increases cortical excitability and improves motor learning in healthy volunteers

**DOI:** 10.1186/s12984-020-00665-7

**Published:** 2020-02-19

**Authors:** Tomofumi Yamaguchi, Kouhei Moriya, Shigeo Tanabe, Kunitsugu Kondo, Yohei Otaka, Satoshi Tanaka

**Affiliations:** 1 Department of Physical Therapy, Yamagata Prefectural University of Health Sciences, 260 Kamiyanagi, Yamagata-shi, Yamagata, 990-2212 Japan; 2 Laboratory of Psychology, Hamamatsu University School of Medicine, 1-20-1 Handayama, Higashi-ku, Hamamatsu, Shizuoka, 431-3192 Japan; 3 Laboratory for Rehabilitation, Tokyo Bay Rehabilitation Hospital, 4-1-1 Yatsu, Narashino, Chiba, 275-0026 Japan; 4 Faculty of Rehabilitation, School of Health Sciences, Fujita Health University, 1-98 Dengakugakubo, Kutsukake, Toyoake, Aichi 470-1192 Japan; 5 Department of Rehabilitation Medicine I, School of Medicine, Fujita Health University, 1-98 Dengakugakubo, Kutsukake, Toyoake, Aichi 470-1192 Japan

**Keywords:** Transcranial direct current stimulation (tDCS), Attention, Cognition, Plasticity, Motor learning, Primary motor cortex (M1), Upper extremity, Rehabilitation

## Abstract

**Background:**

Transcranial direct current stimulation (tDCS) is a non-invasive brain stimulation technique that has the potential to induce motor cortical plasticity in humans. It is well known that motor cortical plasticity plays an essential role in motor learning and recovery in patients with stroke and neurodegenerative disorders. However, it remains unclear how cognitive function influences motor cortical plasticity induced by tDCS. The present study aimed to investigate whether anodal tDCS combined with attention to a target muscle could enhance motor cortical plasticity and improve motor learning in healthy individuals.

**Methods:**

Thirty-three healthy volunteers were assigned to two experiments. In experiment 1, there were three interventional conditions: 1) anodal tDCS was applied while participants paid attention to the first dorsal interosseous (FDI) muscle, 2) anodal tDCS was applied while participants paid attention to the sound, and 3) anodal tDCS was applied without the participants paying attention to the FDI muscle or the sound. Anodal tDCS (2 mA, 10 min) was applied over the primary motor cortex (M1). Changes in motor evoked potentials (MEPs), short-interval intracortical inhibition (SICI), and intracortical facilitation (ICF) were assessed before and immediately after (0 min), and then 10 min, 30 min, and 60 min after each intervention. In experiment 2, we investigated whether the combination of anodal tDCS and attention to the abductor pollicis brevis (APB) muscle could facilitate the learning of a ballistic thumb movement.

**Results:**

Anodal tDCS increased cortical excitability in all conditions immediately after the stimulation. Significant increases in MEPs and significant decreases in SICI were observed for at least 60 min after anodal tDCS, but only when participants paid attention to the FDI muscle. In contrast, no significant changes in ICF were observed in any condition. In experiment 2, the combination of tDCS and attention to the APB muscle significantly enhanced the acquisition of a ballistic thumb movement. The higher performance was still observed 7 days after the stimulation.

**Conclusions:**

This study shows that anodal tDCS over M1 in conjunction with attention to the target muscle enhances motor cortex plasticity and improves motor learning in healthy adults. These findings suggest that a combination of attention and tDCS may be an effective strategy to promote rehabilitation training in patients with stroke and neurodegenerative disorders.

**Trial registration:**

Retrospectively registered (UMIN000036848).

## Background

Transcranial direct-current stimulation (tDCS) is a non-invasive cortical stimulation technique that has the potential to alter cortical excitability [1, 2]. tDCS has also been shown to modulate motor performance and learning in healthy adults, patients with stroke, and patients with neurodegenerative disorders [3–10]. tDCS, therefore, enables the investigation of the causal relationship between local brain activity and behavior as a tool of basic human neuroscience, and also represents a potential new rehabilitation strategy to improve motor function in patients with stroke and neurodegenerative disorders.

However, recent studies have shown that the effects of tDCS are highly variable between studies as well as between individuals [11–14]. While a systematic review pointed out that tDCS has a reliable effect on motor evoked potentials (MEPs), the authors also reported that the magnitude of the effect differs significantly between studies [15]. This inconsistency in effects was further confirmed in other studies that investigated the inter- and intra-individual variability of tDCS in healthy individuals. Facilitation of MEPs was only observed in 45–50% of participants when anodal tDCS was applied to the hand primary motor cortex (M1) [12, 16]. The development of more effective tDCS protocols is thus necessary.

A previous study has shown that cortical plasticity induced in the hand M1 is strongly altered by attention to the target hand [17]. In this study, paired associative stimulation (PAS), a combination of TMS and peripheral nerve stimulation, was applied to M1 and the median nerve, and participants’ attention was manipulated by asking them to either attend to the hand being stimulated or attend away from it by actively engaging in an irrelevant visual task [17]. The results showed that the PAS-induced cortical plasticity of the hand motor cortex was highly enhanced by attention to the target hand, suggesting that attention is a major determinant of motor cortical plasticity. As it remains unknown how attention influences motor cortical plasticity and motor learning induced by tDCS, here, we investigated whether tDCS combined with attention to the target muscle can also enhance cortical plasticity and motor learning in healthy individuals.

## Methods

### Participants

Thirty-three healthy volunteers participated in this study, nine (five women) with a mean age of 25.6 years (standard deviation: SD, 2.7 years) in experiment 1, and 24 (12 women) with a mean age of 24.8 years (SD, 2.4 years) in experiment 2. The sample size was determined on the basis of previous studies investigating the effect of attention on motor cortical plasticity [17, 18]. Each participant’s dominant hand was established using the Chapman’s dominant hand test [19]. All participants were right-handed. None of the participants had a history of neurological disease or were receiving any medication affecting the central nervous system. The participants provided written informed consent prior to participation. The study was approved by the Institutional Review Board of Tokyo Bay Rehabilitation Hospital, Japan, and conformed to the standards set by the latest revision of the Declaration of Helsinki. The study was not pre-registered, because pre-registration was not common in the field of human neurophysiology at the time when the study was conducted, that is, from 2012 to 2014.

### General experimental procedure

Two experiments were conducted in order to investigate the combined effect of tDCS and attention to the target muscle on motor cortical plasticity (experiment 1) and motor skill learning (experiment 2). The methods for each experiment are described in detail below.

### Experiment 1 (neurophysiological experiment)

### tDCS

tDCS was delivered with a DC-Stimulator-Plus (NeuroConn, Ilmenau, Germany) connected to a pair of sponge-surface electrodes soaked in a 0.9% NaCl saline solution. The stimulation duration was set to 10 min. The current was ramped up to 2 mA over a 15-s period and a descending current ramp was used at the end of the stimulation period. The anodal electrode (25 cm^2^) was positioned over the left-hand M1. The location of the hand M1 was determined based on the induction of the largest MEPs in the right first dorsal interosseous (FDI) muscle evoked with TMS. The reference electrode (50 cm^2^) was placed over the ipsilateral upper arm [20–22] in order to minimize the possibility that cathodal stimulation (reference electrode) created unwanted changes in frontal cortex excitability [20, 23]. The current density was 0.08 mA/cm^2^, and the total surface charge density was 0.048 C/cm^2^, both well below the threshold for tissue damage [24].

### Manipulation of attention

The participants were comfortably seated in front of a table in a quiet room. Their hands were covered with a box in all conditions, to avoid visual attention to the target muscle. Participants were asked to fixate on a marker centered in front of them throughout the task.

In order to experimentally manipulate participants’ attention, they were asked to perform a target detection task with a sensory stimulus. During tDCS application, participants were presented with stimuli of two sensory modalities (i.e., somatosensory and auditory stimuli). As the somatosensory stimulus, a weak electric pulse was delivered to the skin just above the right FDI muscle. The pulse duration was 1 ms and the stimulus intensity was 1.1 times the perceptual threshold for each participant. As the auditory stimulus, a beep sound was presented through a headphone. The auditory stimulus intensity was 1.1 times the perceptual threshold for each participant. Both stimuli were presented 20 times at semi-random intervals every 30 s. Participants were asked to verbally report the detection of the sensory stimulus as soon as they detected it. In the somatosensory attention condition, participants detected only the somatosensory stimulus and were to ignore the sound stimulus, whereas in the auditory attention condition, the task was reversed. In order to detect the sensory stimulus, participants needed to pay selective attention to the right FDI muscle (“Attention to Target Muscle” condition) or the beep sound (“Attention to Sound” condition), because the stimuli were just above their sensory threshold and difficult to detect without attention. Participants did not receive any feedback. Error reactions were defined as missed responses (no reaction during stimulation) and incorrect responses (reaction without stimulation). All stimulus conditions and error reaction data are presented in Supplemental data 1.

### Electromyography

Prior to electrode attachment, the skin areas were rubbed with alcohol, and skin resistance was kept below 5 kΩ. Surface electrodes were placed on the right FDI, the abductor pollicis brevis (APB) muscle, and the extensor carpi radialis (ECR) muscle. The raw signal was amplified and filtered (band pass 5–3000 Hz) using a bioelectric amplifier (Neuropack MEB-2200; Nihon Kohden Corp., Tokyo, Japan), digitized at 4000 Hz, and stored for offline analysis on a laboratory computer (Power Lab system; AD Instruments Pty Ltd., New South Wales, Australia).

### Transcranial magnetic stimulation

TMS was delivered using a Magstim 200 stimulator connected through a BiStim module (Magstim Co., Dyfed, UK) to a figure-eight shaped coil with an internal wing diameter of 9 cm. The magnetic stimulator was capable of delivering a magnetic field of 2.2 T for a 100-μs pulse. The coil was placed with the handle pointing backwards, laterally at 45° from the midline, and approximately perpendicular to the central sulcus.

The stimulating coil was placed over the site that was optimal for eliciting responses in the right FDI. The threshold was determined while the FDI was at rest and during voluntary contraction. The threshold was defined as the minimum stimulus intensity that evoked responses of 50 μV with a similar shape and latency during five out of 10 successive stimuli. Each participant was asked to relax during the measurement of the resting motor threshold (rMT) while electromyogram silence was monitored. The active motor threshold (aMT) was defined as the lowest stimulus intensity needed to produce MEPs greater than 200 μV in at least five out of 10 successive trials during the maintenance of 100 μV of FDI voluntary isometric contraction. Although parameters were adjusted for the right FDI (target muscle), the APB and ECR were simultaneously recorded to investigate whether regional effects on motor cortical plasticity were observed when participants paid attention to the target muscle.

The stimulation intensity was set at 120% rMT to assess changes in motor cortex excitability. TMS trials were randomly delivered 15 times, and 15 MEPs were recorded for each time point. Peak-to-peak MEP amplitudes were averaged, and MEP responses were expressed as percentages of experimental MEPs relative to baseline (%MEP).

In order to induce short-interval intracortical inhibition (SICI) and intracortical facilitation (ICF), we applied sub-threshold conditioning paired-pulse stimulation [25]. We used 80% aMTs for the conditioning stimulus and 120% rMTs for the test stimulus. Throughout the experiment, the test stimulus was adjusted to maintain the MEP amplitude equal to the FDI MEP amplitude at baseline. The interstimulus intervals were set at 2 ms (SICI_2ms_) and 3 ms (SICI_3ms_), and at 10 ms (ICF_10ms_) and 15 ms (ICF_15ms_), and 15 MEPs were recorded from the FDI muscle for each ISI and test stimulation. The conditioned MEP amplitudes were expressed as percentages of the mean test MEP amplitudes. The time between stimulus pulses was varied between 5 and 7 s in order to avoid repetitive TMS effects. The stimulus timing was automatically controlled using LabVIEW (National Instruments, Austin, TX, USA).

### Experimental procedure

**Fig. 1:**
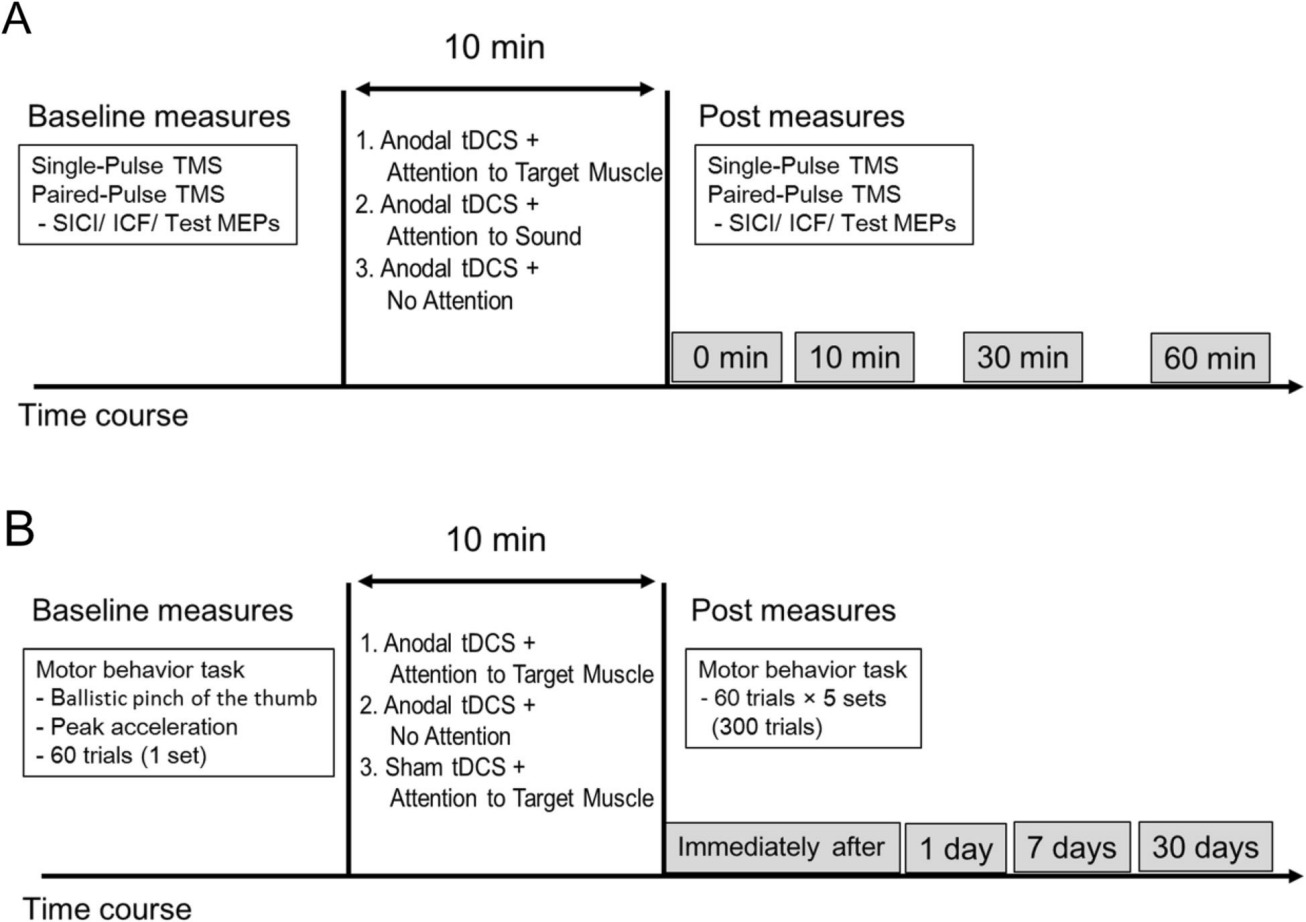
Experimental paradigm for anodal tDCS combined with attention. Time course of experiment 1 (**a**) and experiment 2 (**b**)

The present study employed an assessor-masked randomized crossover design, and all participants performed the following three conditions on different days: 1) anodal tDCS was applied while participants paid attention to the target FDI muscle (anodal tDCS + Attention to Target Muscle), 2) anodal tDCS was applied while participants paid attention to the sound (anodal tDCS + Attention to Sound), and 3) anodal tDCS was applied without the participants paying attention to the FDI muscle or the sound (anodal tDCS + No Attention) (Fig. 1a). The order of the conditions was counter-balanced across participants.

In all conditions, anodal tDCS was applied to the motor cortex of the FDI. In the anodal tDCS + Attention to Target Muscle and the anodal tDCS + Attention to Sound conditions, participants performed a somatosensory or auditory target detection task, respectively, during tDCS. In the anodal tDCS + No Attention condition, participants did not perform a target detection task and were asked to ignore the target FDI muscle or the sound during tDCS.

Changes in MEP, SICI, and ICF were assessed before and immediately after (0 min), as well as 10 min, 30 min, and 60 min after the task. To prevent carry-over effects from previous interventions, washout intervals of 1 week or more were inserted between sessions. Based on previous findings that the manipulation of attention combined with PAS or sensory input increases cortical excitability and reduces intracortical inhibition [21, 22], we hypothesized that anodal tDCS + Attention to Target Muscle would increase MEPs and reduce SICI only in the target FDI muscle, and that the effects would be more prominent and persist longer than the effects in other conditions.

### Complementary experiment

We did not include a sham tDCS condition in experiment 1. It remained unclear whether the significant increase in MEPs after tDCS in the condition where participants paid attention to the target FDI muscle was due to an interaction of tDCS and attention or whether it was an effect of attention alone. To address this question, another eight participants took part in a control experiment with two interventional conditions on different days: 1) sham tDCS + Attention to Target Muscle and 2) sham tDCS + Attention to Sound. The experiment had a double-blind sham-controlled design. Changes in MEPs of the FDI muscle were assessed before and immediately after (0 min), as well as 10 min, 30 min, and 60 min after the task. Mann-Whitney U-tests with Bonferroni adjustments were conducted to evaluate differences in MEPs between the sham tDCS + Attention to Target Muscle and anodal tDCS + Attention to Target Muscle conditions, and between the sham tDCS + Attention to Sound and anodal tDCS + Attention to Sound conditions at each testing time.

### Experiment 2 (behavioral experiment)

#### tDCS

The parameters for tDCS applied to M1 were the same as in experiment 1. The anodal electrode was positioned over the right M1 of the APB, and the reference electrode was placed over the ipsilateral upper arm. For the sham condition, the intensity was set to 2 mA, but the current was applied for only 30 s in order to mimic the sensation of the ramped-up and ramped-down current applied at the end of the anodal condition.

#### Manipulation of attention

Participants performed a somatosensory detection task, with the setting identical to that in experiment 1. During 10 min of real or sham tDCS, participants were presented with a somatosensory stimulus to the APB of the non-dominant left hand at semi-random intervals, about every 30 s, and asked to report when they detected the stimulus.

#### Motor task

A ballistic flexion movement task was used as the practice task, because it has repeatedly been reported that motor skill learning of this task is modulated by repetitive TMS and tDCS [26–29]. Therefore, the target muscle was changed from the FDI muscle in experiment 1 to the APB muscle in experiment 2, because the APB muscle plays an essential role in the ballistic flexion movement task. The forearm was fixed in a neutral position between pronation and supination with the thumb free to move, whereas the fingers were fixed in place with a rigid brace. An accelerometer was then attached to the left thumb pad. The peak acceleration of ballistic thumb movement was recorded with the accelerometer using integral electronics (model 25A; Endevco, San Juan Capistrano, CA, USA). The signal was amplified by a battery-powered, low-noise signal conditioner (model 4416B Isotron Signal Conditioner; Endevco). Acceleration signals were amplified (10×) and digitized at 2000 Hz using an analog-digital converter and recorded on a computer for offline analysis. A customized LabVIEW program was created for triggering movement onset with an auditory signal, providing visual feedback, and recording the motor performance data.

The participants were seated in front of a computer screen. They were asked to flex their left thumb as quickly as possible following a beep sound, and then to completely relax the left hand until the next beat. Acceleration signals were measured for 1.5 s after the auditory signal. At 1.5 s after the accelerometer value was obtained, the participants received visual feedback regarding the peak acceleration of their ballistic thumb movement via a computer screen that presented a color signal. When participants performed faster than the median of the previous five acceleration values, a blue rectangle was presented on the computer screen. In contrast, when participants performed slower than the median of the previous five acceleration values, a red rectangle was presented. Peak acceleration of the ballistic thumb movement was analyzed as an indicator of motor performance. The median value of peak accelerations in each block was calculated.

#### Experimental procedure

We employed a double-blind sham-controlled experimental design. The participants were randomly allocated to one of three groups: 1) anodal tDCS + Attention to Target Muscle, 2) anodal tDCS + No Attention, and 3) sham tDCS + Attention to Target Muscle (Fig. 1b).

Before the intervention, the participants practiced 20 trials of ballistic thumb movements in order to get used to the task. Following that, participants performed one session of the ballistic task (60 trials) as a baseline. After the intervention, they performed five sessions of the ballistic task (300 trials total). Follow-up measurements (five sessions of the ballistic task) were conducted at 1 day, 7 days, and 30 days after the first ballistic task to examine long-term differences in motor performance between the groups. We hypothesized that the enhancement of cortical plasticity induced by anodal tDCS + Attention to the target APB muscle would improve motor learning of the ballistic thumb movement and thus lead to higher long-term performance, compared with the other conditions [17, 18].

#### Statistical analysis

The Shapiro-Wilk test was used to determine whether MEP amplitudes, %MEP, SICI, ICF, and performance data were normally distributed. For experiment 1, a repeated-measures mixed-model analysis of variance (ANOVA) was used to assess the effects of each task (anodal tDCS + Attention to Target Muscle, anodal tDCS + Attention to Sound, anodal tDCS + No Attention) and each testing time (Post0, Post10, Post30, and Post60) on %MEP, SICI, and ICF when the data were normally distributed. Paired t-tests with Bonferroni adjustments for multiple comparisons were performed for post hoc comparisons. For the data that were not normally distributed, the Kruskal-Wallis test was used to assess the main effect of each task (anodal tDCS + Attention to Target Muscle, anodal tDCS + Attention to Sound, anodal tDCS + No Attention) at each time point. Mann-Whitney U-tests with Bonferroni adjustments were conducted to evaluate between-group differences.

For experiment 2, a repeated-measures mixed-model ANOVA with the factors group (anodal tDCS + Attention to Target Muscle, anodal tDCS + NO Attention, sham tDCS + Attention to Target Muscle) and session (baseline, 1 set, 2 sets, 3 sets, 4 sets, and 5 sets of the motor task) was performed to investigate whether the effects of anodal tDCS combined with attention to the APB muscle can enhance the acquisition of ballistic thumb movements. A repeated-measures mixed-model ANOVA with the factors group (anodal tDCS + Attention to Target Muscle, anodal tDCS + No Attention, sham tDCS + Attention to Target Muscle) and time course (baseline, immediate after, 1 day after, 7 days after, 30 days after the motor task) was also performed to test whether the effects of anodal tDCS combined with anodal tDCS can enhance the performance of the learned movement. Multiple pairwise comparisons with Bonferroni adjustments were performed for post hoc comparisons when a significant result was obtained in the primary analyses. For the data that were not normally distributed, Mann-Whitney U-tests with Bonferroni adjustments were performed to evaluate within- and between-group differences. *P* values < 0.05 were considered statistically significant for all analyses. Statistical analyses were performed using IBM SPSS 24.0 (IBM Corp., New York, NY, USA) for Windows.

Data of one participant were missing due a device issue in the sham tDCS + Attention to Target Muscle condition at 1 day after the first ballistic task. There were also some missing data in the anodal tDCS + Attention to Target Muscle (two participants) condition, the sham tDCS + Attention to Target Muscle (three participants) condition, and the anodal tDCS + No Attention (three participants) condition at 30 days after the first ballistic task, due to the same issue.

### Results

The Shapiro-Wilk test confirmed that all data except the MEP amplitudes and %MEP were normally distributed.

### Experiment 1

#### MEP

The mean raw values (standard deviation: SD) of the MEP amplitudes in the FDI muscle at baseline were 0.47 (0.18) mV in the anodal tDCS + Attention to Target Muscle, 0.59 (0.29) mV in the anodal tDCS + Attention to Sound, and 0.57 (0.45) mV in the anodal tDCS + No Attention condition. These baseline values did not significantly differ from each other (Kruskal-Wallis test, *P* = 0.314). The mean raw values (SD) of the MEP amplitudes in the APB at baseline were 0.36 (0.42), 0.43 (0.37), and 0.28 (0.27) mV, also not significantly different from each other (Kruskal-Wallis test, *P* = 0.546). The mean raw values (SD) of the MEP amplitudes in the ECR at baseline were 0.32 (0.30), 0.45 (0.28), and 0.31 (0.21) mV, also not significantly different from each other (Kruskal-Wallis test, *P* = 0.447).

**Fig. 2:**
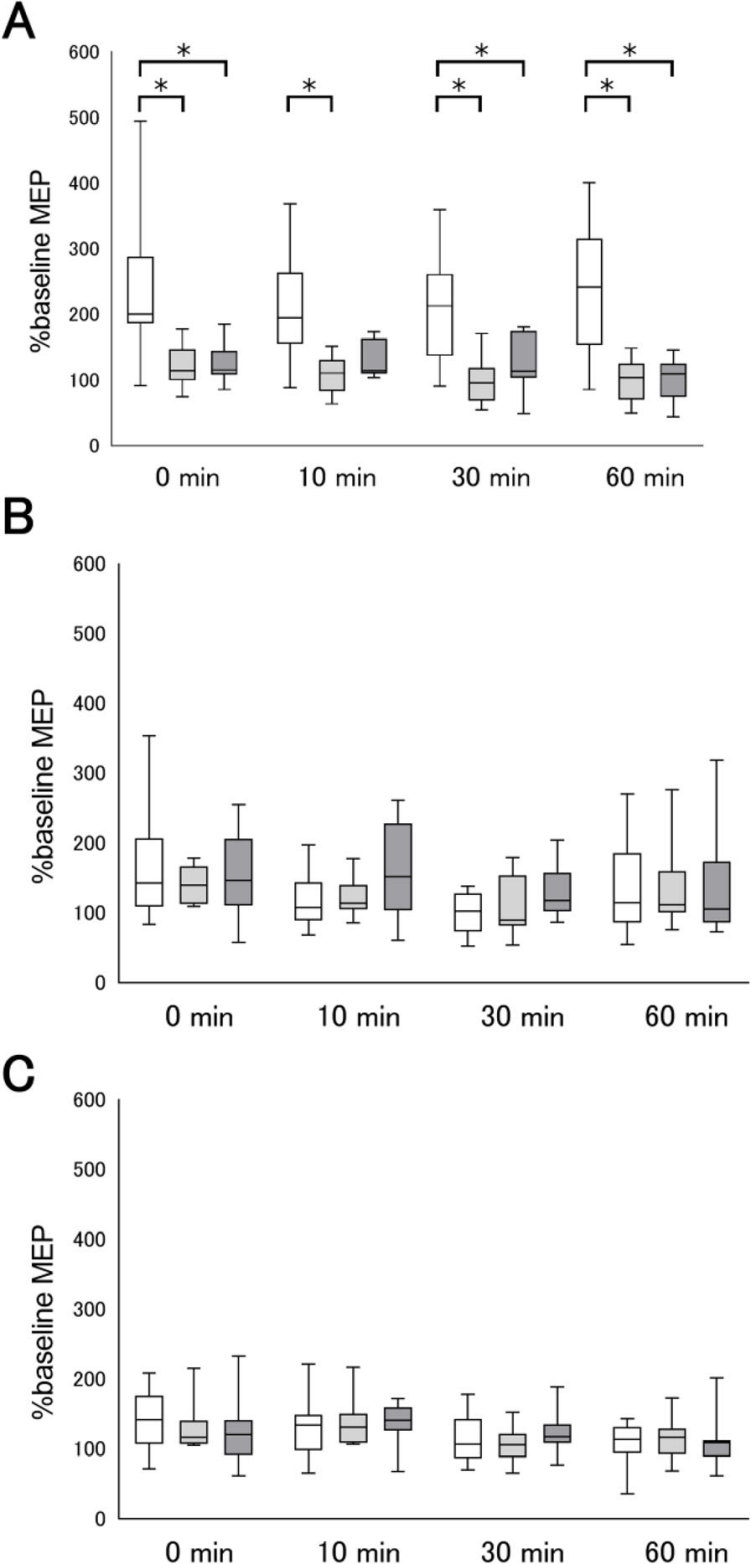
The effects of anodal tDCS combined with attention on motor evoked potentials (MEPs). MEP amplitudes at the first dorsal interosseous (FDI; **a**), abductor pollicis brevis (APB; **b**), and extensor carpi radialis (ECR; **c**) muscles were normalized to the baseline amplitude (%) for each condition. White box plots denote anodal tDCS applied while participants paid attention to the target FDI muscle. Light gray box plots denote anodal tDCS applied while participants paid attention to the sound. Dark gray box plots denote anodal tDCS applied without participants paying attention to the target FDI muscle or the sound. Median and interquartile ranges are represented by horizontal lines within boxes and whiskers (representing minimum and maximum values), respectively. Asterisks indicate significant differences (*P* < 0.05) among the interventions

The time course of the %MEP are shown in Fig. 2. To confirm the effects of anodal tDCS on MEP amplitudes between baseline and Post0 in each muscle (FDI, APB, and ECR), Wilcoxon one-tailed signed rank tests were performed within each condition based on the assumption that anodal tDCS increases MEP amplitudes [7]. Compared to the baseline, MEP amplitudes significantly increased at Post0 in the anodal tDCS + Attention to Target Muscle (*P* = 0.002 for FDI muscle, *P* = 0.004 for APB muscle, and *P* = 0.048 for ECR muscle), in the anodal tDCS + Attention to Sound (*P* = 0.049 for FDI muscle, *P* = 0.039 for APB muscle, and *P* = 0.002 for ECR muscle), and in the anodal tDCS + No Attention condition (*P* = 0.048 for FDI muscle, *P* = 0.004 for APB muscle, and *P* = 0.004 for ECR muscle). These results indicate that anodal tDCS increases cortical excitability in all muscles immediately after the stimulation.

Kruskal-Wallis tests were then used to assess the effects of each task at each time point. There were significant main effects of task on %MEP in the FDI muscle at Post0 (*P* = 0.002), Post10 (*P* = 0.002), Post30 (*P* = 0.004), and Post60 (*P* = 0.003) (Fig. 2a). No significant main effects on %MEP in the APB muscle were found at Post0 (*P* = 0.344), Post10 (*P* = 0.448), Post30 (*P* = 0.118), and Post60 (*P* = 0.798) (Fig. 2b), and no significant main effects on %MEP in the ECR muscle at Post0 (*P* = 0.615), Post10 (*P* = 0.162), Post30 (*P* = 0.927), and Post60 (*P* = 0.395) (Fig. 2c). We found that anodal tDCS + Attention to Target Muscle significantly increased the %MEP in the FDI muscle compared to anodal tDCS + Attention to Sound at Post0 (*P* = 0.008), Post10 (*P* = 0.007), Post30 (*P* = 0.019), and Post60 (*P* = 0.030) (Fig. 2a). In addition, anodal tDCS + Attention to Target Muscle significantly increased the %MEP in the FDI muscle compared to anodal tDCS + No Attention at Post0 (*P* = 0.045), Post30 (*P* = 0.033), and Post60 (*P* = 0.047) (Fig. 2a). These results indicate that attention to the target muscle enhanced the tDCS-induced motor cortical excitability, and the regional effects were observed in the target muscle.

**Fig. 3:**
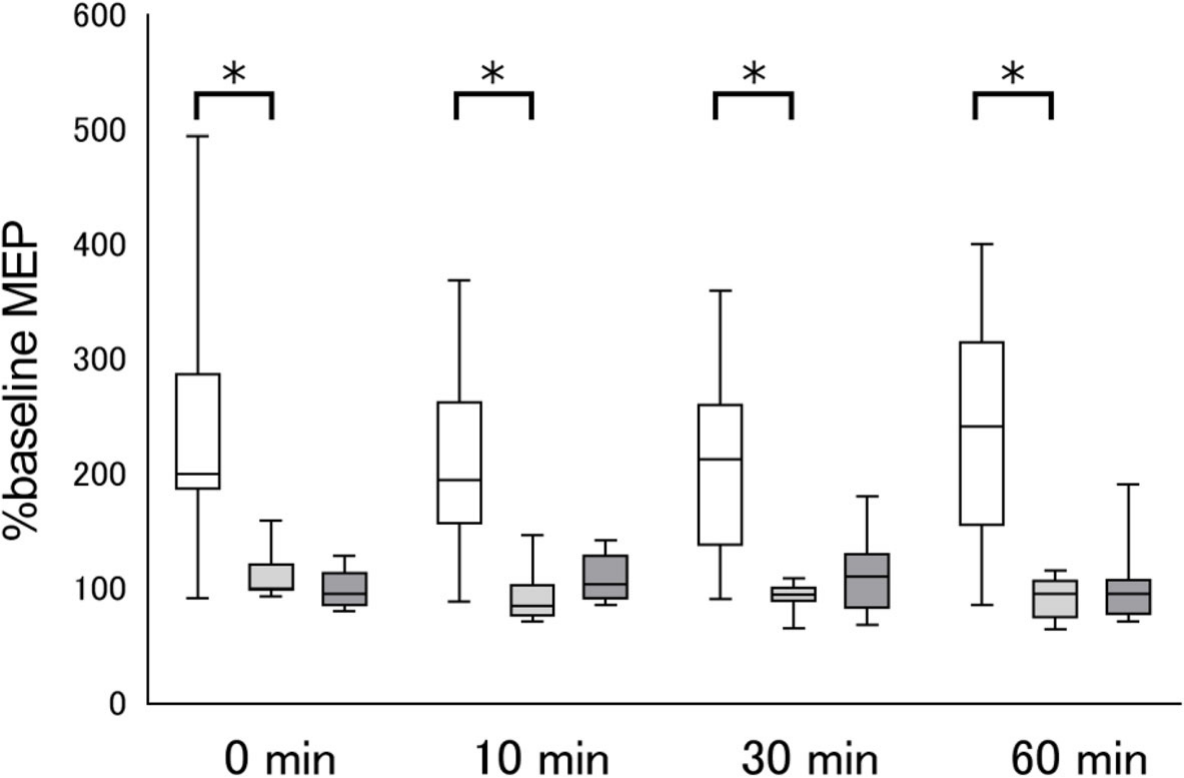
The effects of sham tDCS combined with attention on motor evoked potentials (MEPs). MEP amplitudes at the first dorsal interosseous (FDI) were normalized to the baseline amplitude (%) for each condition. White box plots denote anodal tDCS applied while participants paid attention to the target FDI muscle. Light gray box plots denote sham tDCS applied while participants paid attention to the target FDI muscle. Dark gray box plots denote sham tDCS applied while participants paid attention to the sound. Median and interquartile ranges are represented by horizontal lines within boxes and whiskers (representing minimum and maximum values), respectively. Asterisks indicate significant differences (*P* < 0.05) between anodal tDCS combined with attention to the target muscle and sham tDCS combined with attention to the target muscle. No significant difference was observed between sham tDCS conditions (*P* > 0.05)

The results of the complementary experiment show that anodal tDCS + Attention to Target Muscle significantly increased the %MEP in the FDI at Post0 (*P* < 0.001), Post10 (*P* < 0.001), Post30 (*P* = 0.004), and Post60 (*P* = 0.008) when compared to sham tDCS + Attention to Target Muscle (Fig. 3). There were no significant differences in main effects of task between anodal tDCS + Attention to Sound and sham tDCS + Attention to Sound regarding %MEP in the FDI at Post0 (*P* = 0.321), Post10 (*P* = 0.236), Post30 (*P* = 0.963), and Post60 (*P* = 0.423). These results indicate that motor cortical excitability was only enhanced when anodal tDCS was combined with attention to the target muscle.

#### SICI and ICF

The SICI and ICF values are shown in Table 1. SICI decreased after anodal tDCS combined with attention to the target FDI muscle for 60 min or longer. In contrast, lasting effects were seen for up to 15 min after anodal tDCS without attention. No apparent changes in SICI_2ms_ and SICI_3ms_ were observed in the anodal tDCS + Attention to Sound condition. When SICI was compared between conditions at each testing time point, anodal tDCS + Attention to Target Muscle was found to enhance the modulatory effect of anodal tDCS on SICI_2ms_ and SICI_3ms_. For the ICF, no changes were observed in any of the anodal tDCS conditions.

**Table 1:** Changes in SICI and ICF before and after anodal tDCS combined with attention

	Baseline	0 min	10 min	30 min	60 min
Anodal tDCS + Attention to Target Muscle					
SICI_2ms_	59.6 (8.2)∗	81.4 (8.3)∗,†	79.3 (6.4)∗,†	74.7 (7.5)†, §	71.5 (10.6)∗,†,§
SICI_3ms_	60.2 (11.4)∗	79.1 (7.9)∗	81.0 (7.1)∗	76.1 (8.5)‡	72.4 (9.1)∗,‡
ICF_10ms_	156.0 (45.7)	158.2 (54.2)	166.0 (51.3)	161.8 (57.8)	149.2 (36.8)
ICF_15ms_	169.1 (45.5)	181.0 (83.6)	192.6 (75.8)	156.3 (30.2)	141.5 (24.0)
Test MEP (mV)	0.53 (0.25)	0.59 (0.36)	0.60 (0.34)	0.57 (0.35)	0.55 (0.41)
Anodal tDCS + Attention to Sound					
SICI_2ms_	54.9 (11.2)	62.2 (15.6)†, ||	57.8 (19.5)†	56.1 (15.8)†	57.1 (15.8)†
SICI_3ms_	56.7 (13.4)	61.2 (14.7) ¶	63.7 (20.6)	58.3 (11.9)‡	54.1 (10.0)‡
ICF_10ms_	152.5 (31.2)	138.1 (41.0)	154.0 (46.0)	135.3 (25.2)	131.9 (37.6)
ICF_15ms_	160.5 (42.2)	143.3 (32.7)	140.9 (28.6)	131.2 (16.5)	128.8 (19.5)
Test MEP (mV)	0.66 (0.32)	0.65 (0.33)	0.62 (0.20)	0.64 (035)	0.67 (0.30)
Anodal tDCS + No Attention					
SICI_2ms_	62.6 (12.0)	74.0 (7.6) ||	72.6 (8.8)	64.0 (8.7) §	56.9 (14.0) §
SICI_3ms_	59.4 (10.9)∗	75.8 (9.9)∗, ¶	75.3 (9.7)∗	63.7 (8.1)	59.1 (11.5)
ICF_10ms_	150.9 (44.2)	175.7 (75.7)	171.2 (73.2)	159.2 (51.7)	148.3 (36.6)
ICF_15ms_	144.0 (35.5)	184.1 (84.7)	188.0 (56.1)	143.9 (32.2)	140.9 (34.4)
Test MEP (mV)	0.75 (0.36)	0.70 (0.25)	0.72 (0.34)	0.75 (0.36)	0.69 (0.35)

Values represent mean (standard deviation). Short-interval intracortical inhibition (SICI) and intracortical facilitation (ICF) values represent the percentage of normalized test motor evoked potential (MEP) amplitudes. Asterisks indicate significant differences within conditions when compared to baseline (∗*P <* 0.05). Daggers and double daggers indicate significant differences between anodal tDCS + Attention to Target Muscle and anodal tDCS + Sound at each time point (SICI_2ms_,†*P <* 0.05; SICI_3ms_,‡*P <* 0.05). Section symbols indicate significant differences between anodal tDCS + Attention to Target Muscle and anodal tDCS + No Attention at each time point (SICI_2ms_,§*P <* 0.05). Parallel bars and paragraph symbols indicate significant differences between anodal tDCS + Attention to Sound and anodal tDCS + No Attention at each time point (SICI_2ms_,||*P <* 0.05; SICI_3ms_,¶*P <* 0.05)

The above results were supported by the ANOVAs showing significant interactions between condition and testing time, which were the main factors of interest of the present experiment. The significant main effects of condition (SICI_2ms_: *F*_2_, _16_ = 12.28; SICI_3ms_: *F*_2_, _16_ = 9.51) and testing time (SICI_2ms_: *F*_4_, _32_ = 13.88; SICI_3ms_: *F*_4_, _32_ = 20.86) were qualified by significant interactions for SICI_2ms_ (*F*_8_, _64_ = 2.45) and SICI_3ms_ (*F*_8_, _64_ = 2.12). The interactions indicated that testing time interfered with condition, showing that the effect of condition was mainly attributable to the testing time point on SICI_2ms_ and SICI_3ms_. To clarify this effect, multiple pairwise comparisons were performed for post hoc analysis.

Compared to the baseline values, anodal tDCS + Attention to Target Muscle significantly decreased SICI_2ms_ and SICI_3ms_ at Post0, Post15, and Post60, while anodal tDCS + No Attention significantly decreased SICI_3ms_ at Post0 and Post15 (see Table 1).

SICI_2ms_ was significantly decreased by anodal tDCS + Attention to Target Muscle, compared to anodal tDCS + Attention to Sound at Post0, Post15, Post30, and Post60 (see Table 1). Compared to anodal tDCS + No Attention, SICI_2ms_ was also significantly decreased at Post30 and Post60. SICI_3ms_ was significantly decreased by anodal tDCS + Attention to Target Muscle at Post30 and Post60, compared to anodal tDCS + Attention to Sound. Anodal tDCS + No Attention significantly decreased SICI_2ms_ and SICI_3ms_ at Post0 compared to anodal tDCS + Attention to Sound.

No significant interaction was found for ICF_10ms_ (*F*_8_, _64_ = 0.60) or ICF_15ms_ (*F*_8_, _64_ = 1.12). There were no significant main effects of protocol and testing time for ICF_10ms_ (condition: *F*_2_, _16_ = 1.24; testing time: *F*_4_, _32_ = 0.69) or ICF_15ms_ (condition: *F*_2_, _16_ = 2.29; testing time: *F*_4_, _32_ = 1.70). These results indicate that the effect of condition was not attributable to the testing time point on ICF_10ms_ and ICF_15ms_.

#### Experiment 2

Mean (SD) motor performance at baseline, measured as peak acceleration, was 3.35 (0.51) g in the anodal tDCS + Attention to Target Muscle condition, 3.54 (0.49) g in the anodal tDCS + No Attention condition, and 3.22 (0.61) g in the sham tDCS + Attention to Target Muscle condition. Baseline motor performance was not significantly different among the three conditions (ANOVA, *F*_2_,_24_ = 0.09, *P* = 0.914).

#### Immediate effect on motor learning

**Fig. 4:**
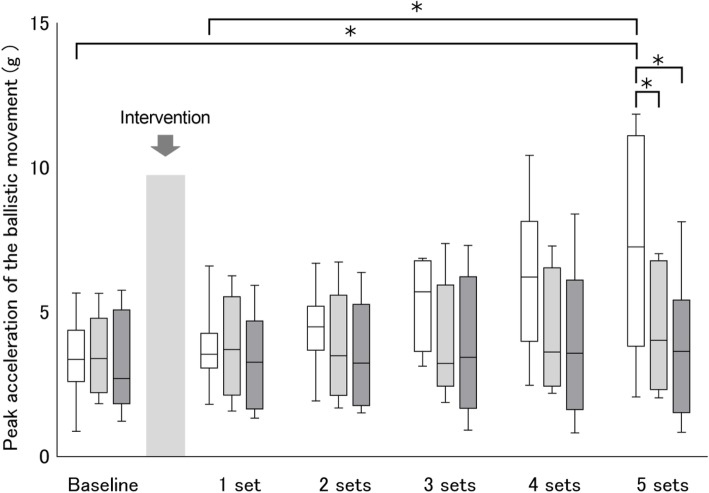
Immediate effects of anodal tDCS combined with attention on motor learning. White box plots denote anodal tDCS applied while participants paid attention to the target APB muscle. Light gray box plots denote anodal tDCS applied without participants paying attention to the target APB muscle. Dark gray box plots denote sham tDCS applied while participants paid attention to the target APB muscle. Median and interquartile ranges are represented by horizontal lines within boxes and whiskers (representing minimum and maximum values), Asterisks indicate significant differences (*P* < 0.05) between the baseline and each intervention time point, or within the interventions

The time course of motor performance in each block after the interventions is shown in Fig. 4. A significant interaction was found for motor performance (*F*_10_,_105_ = 3.54, *P* < 0.001). There was a significant main effect of session (*F*_5_,_105_ = 10.02, *P* < 0.001), while no main effect was found for group (*F*_2_,_21_ = 1.46, *P* = 0.254). Motor performance improved after set 5 in the anodal tDCS + Attention to Target Muscle condition, compared to baseline (*P* = 0.013), and after the first set of the ballistic movement task (*P* = 0.039) (Fig. 4). Performance was significantly improved after set 5 in the anodal tDCS + Attention to Target Muscle condition compared to the anodal tDCS + No Attention (*P* = 0.048) and the sham tDCS +Attention to Target Muscle (*P* = 0.014) condition. This resulted in the anodal tDCS + Attention to Target Muscle group outperforming the other groups following the first set of the ballistic movement task, indicating that anodal tDCS + Attention to Target Muscle applied before a ballistic movement task enhances motor skill acquisition.

#### Changes in performance up to 30 days after interventions

**Fig. 5:**
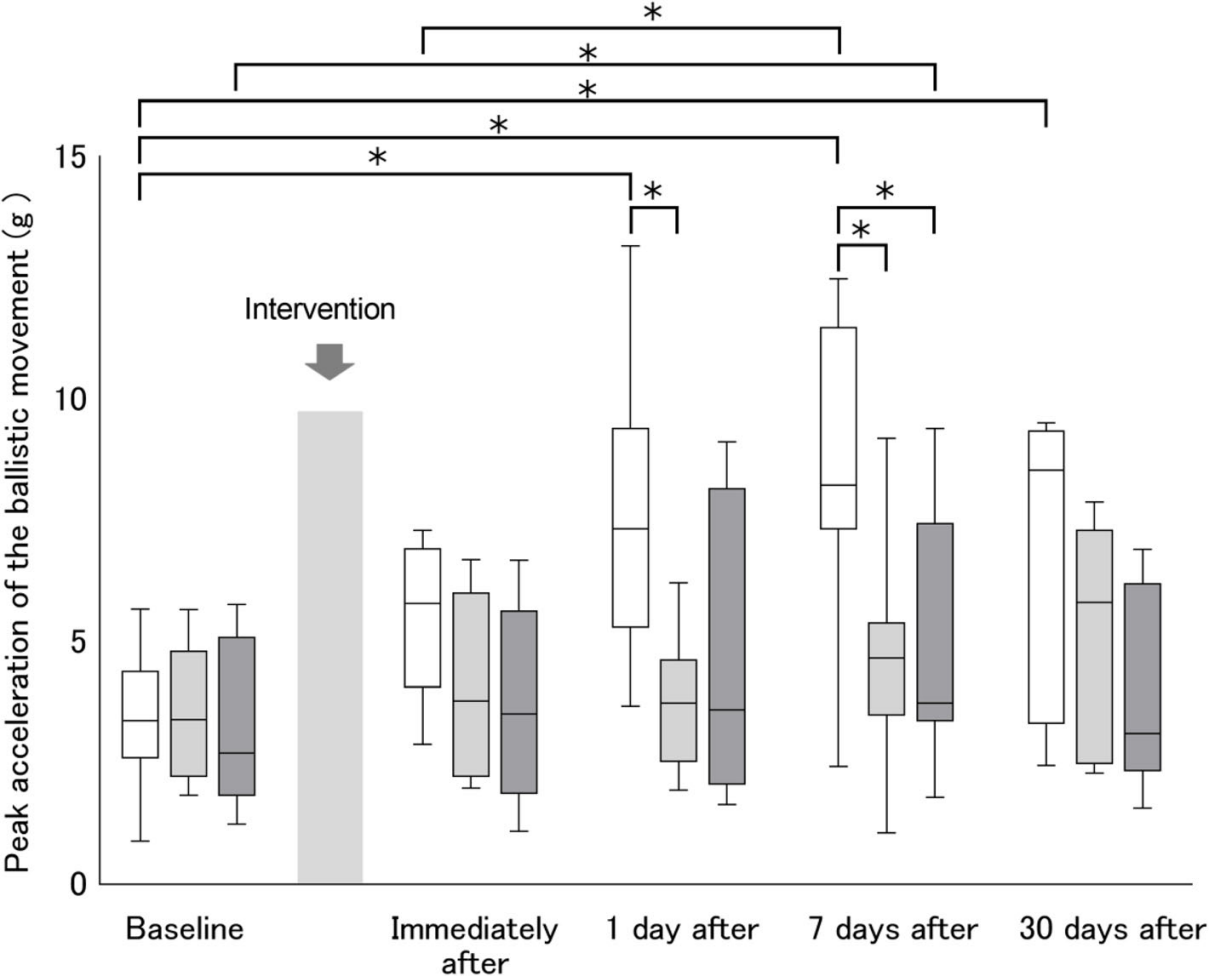
Changes in motor performance up to 30 days after anodal tDCS combined with attention. White box plots denote anodal tDCS applied while participants paid attention to the target APB muscle. Light gray box plots denote anodal tDCS applied without participants paying attention to the target APB muscle. Dark gray box plots denote sham tDCS applied while participants paid attention to the target APB muscle. Median and interquartile ranges are represented by horizontal lines within boxes and whiskers (representing minimum and maximum values), Asterisks indicate significant differences (*P* < 0.05) between the baseline and each intervention time point, or within the interventions

The time course of motor performance on each day after the interventions is shown in Fig. 5. A significant interaction was found for motor performance (*F*_8_,_75_ = 3.31, *P* = 0.003), and significant main effects of group (*F*_2_,_21_ = 3.56, *P* = 0.046) and time course (*F*_4_,_75_ = 13.09, *P* < 0.001). Compared to the baseline values, anodal tDCS + Attention to Target Muscle significantly improved motor performance at 1 day after (*P* = 0.001), 7 days after (*P* < 0.001), and 30 days after the motor task (*P* = 0.012) (Fig. 5). Sham tDCS + Attention to task significantly improved motor performance at 7 days after the motor task (*P* = 0.046), while anodal tDCS + No Attention did not improve performance. Motor performance was significantly increased by anodal tDCS + Attention to Target Muscle (compared to anodal tDCS + No Attention and sham tDCS + Attention to Target Muscle) at 1 day after (vs. anodal tDCS + No attention, *P* = 0.020), and 7 days after (vs. anodal tDCS + No attention, *P* = 0.024; vs. sham tDCS + Attention to Target Muscle, *P* = 0.039) the intervention. These results indicate that anodal tDCS combined with attention to the target muscle enhances the performance of the learned ballistic movement.

## Discussion

In the electrophysiological study (experiment 1), the enhancement of corticospinal excitability and SICI disinhibition was observed only in the FDI muscle when participants paid attention to the FDI muscle during anodal tDCS, even though anodal tDCS increased the corticospinal excitability in all muscles immediately after stimulation. In the behavioral study (experiment 2), the combination of tDCS and attention to the target APB muscle enhanced the learning of the ballistic thumb movement at least 7 days after the intervention.

In experiment 1, the sensory input (sound) and peripheral and cortical electrical stimulation were all identical among the three conditions, and only the participants’ direction of attention was experimentally manipulated. In addition, the additional control experiment shows that attention without real tDCS did not induce significant changes in MEPs. The changes in plasticity may therefore have occurred as a result of an interaction between tDCS and attention, rather than being an effect of attention alone. The present results suggest that participants’ internal mental state, namely attention, is an important factor that determines the effectiveness of tDCS.

In contrast, attention to the sound failed to enhance the effects of anodal tDCS on cortical excitability. Previous studies have indicated that cognitive attention to unrelated activities (e.g., volitional movement during motor imagery) impairs the effects of anodal tDCS on motor cortical excitability [30–32]. Similarly, PAS-induced motor cortical plasticity disappeared when the attention was directed towards the non-target hand or when a cognitive task was presented during the stimulation [17]. Thus, cognitive and attentional load caused by unrelated activities may lead to deactivation of motor cortical excitability, and this may reduce the effect of tDCS on motor cortical plasticity.

A previous TMS study has shown that the after-effects of PAS can be enhanced when participants pay attention to their hand [17]. On the other hand, the present study is the first to report that attention to the target muscle can enhance the effects of anodal tDCS on cortical excitability and motor learning. It has repeatedly been reported that the effect of tDCS largely varies among individuals [11–14] and is relatively small overall [15]. It is therefore important to develop new effective protocols for tDCS. The significance of the present study is that it suggests that a combination of attention and tDCS may be a novel effective approach to promote cortical activity and motor learning. Especially the muscle-selective effect shown in the present study can be a practical advantage when a specific muscle is targeted in clinical and laboratory settings. For example, the combination of tDCS and attention may help improve pinch function in patients with moderate and severe stroke when patients pay attention to the APB or FDI [33]. The pinch strength of the hemiplegic hand is associated with independence in ADLs [34]. Therefore, a combination of tDCS and attention may be an effective way to promote rehabilitative training.

What might be the mechanism underlying the enhancement of cortical excitability by the combination of tDCS and attention? Previous studies suggest that attention modifies neuronal firing rates [35]. Attention to stimuli leads to an increase in the response of sensory neurons to these stimuli. For example, attention to low luminance contrasts increased the responses of V4 neurons in monkeys [36]. In the present study, attention to the target FDI muscle may thus increase the response of motor neurons in M1. Taking into account the increase in excitability induced by anodal tDCS, the additional synaptic activation of motor neurons by top-down attentional systems may lead to synaptic specificity and change synaptic strength. Neurochemically, the cholinergic system is known to contribute to top-down control of attention [37], involving the induction of synaptic plasticity [38–40]. Anodal tDCS promoted increased short latency afferent inhibition (SAI), which can be related to central cholinergic interneuronal circuits [41]. There is also experimental evidence that cholinergic activity facilitates the induction of long-term potentiation (LTP) [42–44]. Thus, the combination of tDCS and attention may modulate cholinergic activity, which enhances synaptic transmission and LTP induction. These mechanisms may be crucial for the plastic changes associated with motor learning and memory formation observed in experiment 2.

In the present study, we also observed a significant decrease in SICI when attention was paid to the FDI muscle. This suggests that suppression of the inhibitory system contributes to the increase in motor cortex excitability. A decrease in SICI is thought to reflect the reduced activity of the GABA-based system in M1 [45]. A previous study reported that anodal tDCS induces the suppression of SICI [46]. In accordance with other SICI studies, a magnetic resonance spectroscopy (MRS) study also reported that anodal tDCS over M1 produced significant reductions in GABA concentration [47–49]. Accordingly, in the present study, attention may have facilitated the suppression of GABAergic inhibitory systems via anodal tDCS. Meanwhile, we found no significant changes in ICF after any of the interventions. Previous studies indicated that the test-retest reliability of ICF is less than that of SICI [50, 51], suggesting that the low reliability may have contributed to the absence of effects on ICF-mediating cortical circuits.

The observed muscle-specific effect could be interpreted in a framework of activity-dependent effects of tDCS [52–54]. There is evidence that DC polarization can induce targeted changes when combined with motor training or synaptic activation [52, 54]. For example, the effect of tDCS is modulated by the timing of motor training [53] and tDCS without training does not improve motor learning [52]. Electrophysiologically, DC stimulation induced long-lasting LTP in mouse M1 slices when combined with repetitive low frequency synaptic activation [52]. In a similar way, we speculate that tDCS may enhance the selective synaptic activation of motor neurons, which is moderated by top-down attention to the target muscle. Our results also suggest that attention could be another mediator that induces activity-dependent effects of tDCS.

This study has several limitations. First, the sample size is small; it was determined on the basis of previous studies, while it should have been based on a power analysis. Second, this study does not represent a pre-registered trial, because pre-registration was not common when we conducted it (2012–2014). Third, since the task used in the present study is a ballistic finger movement, it remains unclear whether our results apply to other motor learning tasks, and whether this approach is also effective for the training of activities of daily living that are essential for rehabilitation; data from our preliminary study does however suggest that the combination of tDCS and attention enhances the performance of skilled hand functions in patients with stroke [33].

## Conclusions

The present study shows that anodal tDCS over M1 significantly enhances motor cortex excitability and improves motor learning and retention of ballistic finger movements when combined with attention to the FDI or APB muscles. Our findings suggest that the combination of attention and tDCS may be an effective way to promote rehabilitation training in patients with stroke and neurodegenerative disorders.

## Supplementary information

**Additional file 1.** Supplemental data 1. Stimulus conditions and error reaction data.

## Abbreviations

AMT: Active motor threshold
ANOVA: Analysis of variance
APB: Abductor pollicis brevis muscle
ECR: Extensor carpi radialis muscle
FDI: First dorsal interosseous
ICF: Intracortical facilitation
ISI: Interstimulus interval
LTP: Long-term potentiation
MEP: Motor evoked potential
MRS: Magnetic resonance spectroscopy
PAS: Paired associative stimulation
RMT: Resting motor threshold
SAI: Short latency afferent inhibition
SICI: Short-interval intracortical inhibition
TDCS: Transcranial direct current stimulation
TMS: Transcranial magnetic stimulation
