# Supplementary material for: Transcranial direct-current stimulation combined with attention increases cortical excitability and improves motor learning in healthy volunteers
Source: J Neuroeng Rehabil. 2020 Feb 19;17:23. doi: 10.1186/s12984-020-00665-7 (PMC7031972; doi:10.1186/s12984-020-00665-7)
Supplement: Supplementary file 1 — Additional file 1. Supplemental data 1. Stimulus conditions and error reaction data. [file 12984_2020_665_MOESM1_ESM.docx]

Supplemental data 1. Stimulus conditions and error reaction data.

|  | Electric intensity [mA] | Sound volume  [dB] | Error reaction [number] | Rate of error reaction[%] |
| --- | --- | --- | --- | --- |
| Anodal tDCS + Attention to Target Muscle (n = 9) | 1.8 (0.6) | 20.3 (4.7) | 1.7 (0.2) ∗ | 8.3 (10.0) ∗ |
| Anodal tDCS + Attention to Sound (n = 9) | 1.5 (0.3) | 18.8 (5.4) | 0.1 (0.3) ∗ | 0.6 (1.7) ∗ |
| Anodal tDCS + No attention (n = 9) | 1.7 (0.6) | 19.1 (3.3) | - | - |
| Sham tDCS + Attention to Target Muscle (n = 8) | 1.3 (0.3) | 22.1 (3.8) | 0.4 (0.5) | 1.9 (2.6) |
| Sham tDCS + Attention to Sound (n=8) | 1.8 (0.4) | 15.0 (5.4) | 0.5 (0.8) | 2.5 (3.8) |

Values represent mean (standard deviation). Asterisks indicate significant differences between anodal tDCS + Attention to Target Muscle and anodal tDCS + Attention to Sound, assessed with paired t-tests (∗*P <* 0.05).
